# Motivation in the Bergen 4-day treatment for obsessive–compulsive disorder

**DOI:** 10.1186/s12888-025-07218-z

**Published:** 2025-10-09

**Authors:** Håvard Berg, Kristian Tjelle, Stian Solem, Bjarne Hansen, Gerd Kvale, Thröstur Björgvinsson, Kristen Hagen

**Affiliations:** 1 Department of Psychiatry, Møre og Romsdal Hospital Trust, Molde Hospital, Molde, 6412 Norway; 2 Department of Psychology, Norwegian University of Science and Technology, Trondheim, Norway; 3 Bergen Center for Brain Plasticity, Haukeland University Hospital, Bergen, Norway; 4 Department of Psychosocial Sciences, University of Bergen, Bergen, Norway; 5 Department of Clinical Psychology, University of Bergen, Bergen, Norway; 6 Behavioral Health Partial Hospital Program, Harvard Medical School, McLean Hospital, Belmont, MA USA; 7 Department of Mental Health, Norwegian University of Science and Technology, Trondheim, Norway

**Keywords:** Treatment engagement, Commitment, Compliance, OCD, CBT, ERP, RCT

## Abstract

**Background:**

This study investigated the associations among treatment motivation, patient adherence, and treatment outcomes in a group of outpatients with difficult-to-treat obsessive–compulsive disorder (OCD).

**Methods:**

A total of 163 relapsed or nonresponding patients were treated with the Bergen 4-day treatment. Motivation was measured with a modified version of the Nijmegen Motivational List 2 (NML2) prior to the start of treatment. During treatment, patients rated their own adherence using the Patient Exposure and Response Prevention (EX/RP) Adherence Scale (PEAS). Treatment outcomes were assessed with the Yale-Brown Obsessive Compulsive Scale (Y-BOCS) at posttreatment and 3-month follow-up.

**Results:**

The NML2 items related to commitment showed a weak but significant association with treatment outcomes but were not significant when patient adherence was controlled for. Higher adherence was strongly associated with better treatment outcomes.

**Discussion:**

Self-reported motivation before the start of treatment had limited predictive validity, whereas in- and between-session patient adherence, which could reflect one aspect of the motivation construct (treatment engagement), was important for recovery.

**Trial registration:**

ClinicalTrials.gov identifier: NCT02656342 (First registered: 2015–11–30).

**Supplementary Information:**

The online version contains supplementary material available at 10.1186/s12888-025-07218-z.

## Introduction

Cognitive behavioral therapy (CBT), which focuses on exposure and response prevention (ERP; [1]), is recommended as the primary psychological treatment for obsessive‒compulsive disorder (OCD). Despite evidence showing that CBT is highly effective in the treatment of OCD [2], there are still treatment challenges that need to be addressed, such as treatment attrition, insufficient response, and relapse [3]. One factor of interest in explaining such phenomena is patient motivation. Treatment motivation may be associated with dropout and treatment outcomes, but systematic reviews of predictor studies have revealed inconsistent findings [4–6], and the motivation construct involves conceptual confusion and ambiguous measures [7].

Motivation has been defined as the probability that a person will enter into, continue, and adhere to a specific change strategy [8], but the definition has received criticism for being circular and ignoring situational factors such as time constraints, financial limitations, or other external barriers [7]. Furthermore, Driechner and colleagues [7] noted that the conceptual ambiguity of measures for treatment motivation can partly be attributed to excessive reliance on exploratory factor analysis and the use of items with a great deal of heterogeneity. Drieschner and colleagues stressed that a rigorous conceptual distinction between treatment motivation, its determinants, and its behavioral consequences is needed. They defined treatment motivation as a patient’s motivation to engage in treatment, which is associated with both treatment engagement and treatment outcomes. The determinants of motivation to engage in treatment include problem recognition, perceived treatment costs, perceived treatment suitability, outcome expectancy, external pressure, and distress. Treatment engagement involves keeping appointments; making sacrifices (financial, social, and psychological) to attend treatment; being open to treatment (not withholding relevant facts and thoughts); actively participating; being constructive both in and between sessions; and being committed to therapy goals.

Despite psychometric and conceptual issues, some early studies with small samples reported that treatment motivation, measured via the original Nijmegen Motivational List (NML), can be used to predict CBT outcomes for patients with OCD [9, 10]. As the NML was criticized for its poor psychometric properties, the Nijmegen Motivational List 2 (NML2) was developed (the NML was later adapted to youth populations and preventive interventions). The first study on the NML2 [11] involved a mixed outpatient group (*N* = 132–124) at different phases of treatment. Of the 34 items, 25 remained after principal component analysis using varimax rotation, which is a method to simplify the interpretation of factor loadings by maximizing the variance of squared loadings across components. The study identified three factors: preparedness, distress, and doubt. However, the small sample size made the study underpowered for factor analysis. Nevertheless, the study revealed that the NML2 total score and the Preparedness subscale score were associated with treatment attrition and patients’ reports of how helpful the sessions were. A similar result was later reported, as low motivation measured via the NML2 (34 items) was associated with dropout in patients receiving CBT for panic disorder, but the association was weak [12].

Other studies on CBT for OCD have used different measures of motivation and assessment times. In one study, University of Rhode Island Change Assessment Scale (URICA; [13]) scores after 4–6 CBT sessions showed a moderate effect on OCD [14]. However, other studies using pretreatment motivation levels as a predictor have reported inconsistent or nonsignificant results. In a study of CBT for OCD using pretreatment URICA scores, patients who dropped out scored lower on the contemplation subscale, but readiness to change was not related to treatment outcomes [15]. The same nonsignificant result for pretreatment URICA scores was found in a related study of CBT for OCD [16]. Nonsignificant findings using the NML2 to predict CBT outcomes have also been reported by studies of mixed anxiety disorders [17] and panic disorder [18].

Crane et al. [19] conducted a systematic review of 22 studies investigating the role of motivational readiness in CBT across mental health conditions. Thirteen studies reported significant associations between readiness and symptom outcomes, but findings were mixed, and associations with treatment attendance were inconclusive. While motivation may influence outcomes, patient adherence could be a more consistent predictor in the context of CBT. Motivation and patient adherence could be related constructs. A recent meta-analysis by Leeuwerik et al. [20] reviewed 123 studies and found that non-adherence is a substantial challenge in CBT for OCD. About 15.6% of eligible patients refused treatment, and 15.9% of those who started CBT dropped out. Adherence to between-session CBT tasks was generally moderate to good and showed a significant medium to large association with symptom reduction. These findings highlight the clinical importance of adherence as a predictor of treatment outcome.

Adherence resembles the concept of treatment engagement [7], especially with respect to actively participating (in this context, exposure and response prevention exercises). Whereas inconsistent findings for pretreatment motivation have been reported, patient adherence (sometimes referred to as compliance) is a reliable predictor of CBT treatment outcomes in patients with OCD [21, 22]. Using data from the same study, higher levels of patient adherence showed a robust association with better treatment outcomes such as OCD symptoms and work and social functioning [23].

It is likely that motivation and adherence fluctuate at different phases of treatment. Ponzini and colleagues [24], using the URICA (assessed at admission, after 1 month of treatment, and at discharge), reported that residential treatment outcomes are predicted by readiness to change and committed action (commitment to behavioral change). Readiness to change was stable (trait-like) across treatment, whereas committed action and OCD severity showed state-like effects. The researchers noted that while patients may enter treatment with high readiness for change, difficulties encountered during therapy may affect their commitment.

In addition to fluctuating motivation throughout treatment, social desirability bias is another issue affecting pretreatment self-report measures of motivation, which could support the need for other ways to assess motivation. A different approach to measuring motivation involves analyzing patients’ in-session statements via the Motivational Interviewing Skill Code (MISC; [25]) framework. One such study on CBT for generalized anxiety disorder reported that patients’ commitment statements in Session 1 and signs of active participation in Session 4 were associated with symptom improvement [26]. Lassen and colleagues [27] also reported that taking steps (recent actions undertaken to challenge their problem) increased more during treatment for treatment responders than for nonresponders. They also noted that taking steps might reflect or overlap with treatment adherence. This was later tested in a subsequent study [28], which revealed a strong association between taking steps and adherence in Session 7 (*r* = .64); however, both constructs were significant predictors of treatment outcomes, suggesting increased predictive power as therapy progressed.

The aim of this study was to investigate whether treatment motivation could predict treatment outcomes in a group of patients with difficult-to-treat OCD (nonresponders or relapsed patients) treated with the Bergen 4-day Treatment (B4DT). No such previous studies exist. The B4DT is a concentrated, exposure-based CBT treatment in which patients are treated over four consecutive days in a combined individual and group setting [29–31]. The B4DT has shown encouraging treatment results in a series of trials [29, 32–38]. The concentrated format of the B4DT ensures reduced influence from external variables and enables more control over the treatment process, thereby making it easier to investigate predictors.

More specifically the study had three main objectives: 1) to assess the reliability of the NML2 measure; 2) to examine the associations between NML2 and relevant outcome measures while controlling for the role of patient adherence; and 3) to establish the validity of cutoff scores for the NML2 and adherence measures cutoff scores in relation to treatment outcome. The main hypothesis was that treatment motivation and adherence would be associated with symptom improvement. We also expected a positive association between motivation and adherence.

## Methods

### Participants and procedure

This study was based on data from a previously published trial testing whether D-cycloserine could potentiate treatment outcomes for patients with difficult-to-treat OCD treated with the B4DT [39]. The original study used a triple-blind, three-armed, placebo-controlled design in which patients within each stratum were randomized to receive 100 mg D-cycloserine, 250 mg D-cycloserine, or placebo at a 2:2:1 ratio. For the current study we did not include treatment condition as a variable because the original study found no differences in treatment outcomes between the three conditions.

Patients were assessed by local OCD teams as part of ordinary clinical practice. They had to meet the criteria for OCD according to the Diagnostic and Statistical Manual of Mental Disorders-Fifth Edition (DSM-5) and have a score of 16 points or more on the Yale-Brown Obsessive Compulsive Scale (Y-BOCS). Patients had to be 18 years old or older, fluent in Norwegian, and capable of receiving treatment in an outpatient clinic. The study consisted of patients who had either not responded to previous ERP treatment or relapsed. The prior ERP treatment had to consist of at least six sessions of documented in-person therapist-guided treatment, ending a minimum of four weeks before inclusion in the study. Relapse was defined as having at least a 35% increase in the Y-BOCS total score and a Clinical Global Impression-Improvement (CGI-I) score of six points (“much worse”) or higher [40]. A nonresponse was defined as having less than a 35% decrease and retaining a score of 16 points or higher on the Y-BOCS. In total, 38.7% of the participants were categorized as nonresponders, whereas 61.3% were categorized as relapsed patients.

Patients were excluded if they met any of the following criteria: current substance abuse/dependence, suicidal ideation or plans, psychosis or bipolar disorder, an unstable antidepressant medication dosage in the last 12 weeks or refusal to maintain a stable dosage during the four treatment days, refusal to refrain from using anxiety-reducing substances (e.g., anxiolytics and alcohol) during the two exposure days, intellectual disability (as indicated by a previous medical history), and a travel time of one hour or more to reach the treatment location. With respect to the administration of D-cycloserine, the exclusion criteria included pregnancy or breastfeeding, renal impairment, hypersensitivity to D-cycloserine, porphyria, and epilepsy. A summary of the sample characteristics is shown in Table 1.

**Table 1:** Patient characteristics before treatment

Characteristic	*M* (*SD*)/% (*n*)
Age	34.6 (10.9)
Male sex	28.2 (46)
Duration of OCD (years)	16.2 (10.2)
Any comorbid disorders	69.3 (113)
Number of comorbid disorders	1.7 (1.9)
Family history of OCD	42.1 (64)
Years in school	11.9 (3.9)
Employment	
Work	34.8 (56)
Student	20.5 (33)
Disability	44.7 (72)
Using any psychotropic medications	46.6 (76)
Using SSRI	31.9 (52)

The percentages are reported as valid percentages in the case of missing values

*OCD* obsessive‒compulsive disorder, *SSRI* selective serotonin reuptake inhibitor

### Treatment

The participants received the B4DT, which is a concentrated four-day, exposure-based CBT treatment [41]. During the first day of the B4DT, the participants are initially taught the key treatment principles in a psychoeducational group with 3–6 participants before they begin planning individually tailored exposure tasks and to their individual treatment goals. In the next two days, the participants actively seek out and engage in anxiety-evoking exposure situations with the assistance of an individual therapist. The main goal of the treatment is to learn new ways to cope with anxiety-eliciting situations without resorting to short-term anxiety-reducing behavior [35]. During the treatment, a therapist helps the participants engage in exposure tasks, identify anxiety-reducing behavior and suggest adaptations to the planned tasks, to further increase the efficiency of each exposure task [42].

The groups come together at prearranged intervals throughout each treatment day to share their experiences, learn from each other and provide support. The participants are expected to continue with exposure tasks after leaving the clinic on Days Two and Three. To ensure progress, the participants are offered phone support at prearranged time points. On Day Three, close relatives are invited to visit the clinic and listen to an abbreviated version of the key treatment principals [41].

Day Four of treatment involves connecting the theory of the treatment with real-life experiences from the previous three days [37]. The key treatment points are summarized while the group is together, and three weeks of self-administered exposure tasks are planned to ensure that the participants are able to continue working on their own.

### Measures

*The NML2* is a self-report form used to assess patient motivation for psychotherapy [11]. The adapted NML2 consists of 25 items and three subscales: Preparedness, Distress, and Doubt. The NML2 was administered to participants as a self-report form prior to the start of treatment. The first version of the NML2 used a 6-point rating scale, but the authors recommended a 5-point scale in further studies [11]. In this study, we used a 5-point scale ranging from 1 (not at all) to 5 (very applicable).

In the original study of the NML2, the Cronbach’s alpha was.81 for the Preparedness subscale,.72 for the Doubt subscale, and.69 for the Distress subscale after one item for each of the factors was removed. The study did not report internal consistency for the total score. The correlations among the three subscales ranged from −.26 to.23. Several studies have tested the validity of the NML2 in different populations; however, different versions of the NML2 (34 items, 25 items, and 24 items) have been used, with some studies using total scores and others using a specific subscale score. Some studies used a 6-point scale rather than a 5-point scale for scoring, and no studies have reported the internal consistency of the NML2 or its subscales [17, 43–45].

The Yale-Brown Obsessive-Compulsive Scale (Y-BOCS; [46]) measures the severity of OCD via a semistructured clinical interview and was used to evaluate treatment outcomes. The Y-BOCS is made up of 10 items scored on a scale ranging from 0–4, which are evenly divided between the obsessions and compulsions dimensions. The Y-BOCS was established as the gold standard for OCD assessment and has good internal consistency (*α* = .89) [46, 47]. Only total scores of the Y-BOCS were used in this study.

*The Patient Health Questionnaire-9* (PHQ-9; [48]) measures depressive symptoms via a self-report questionnaire. The PHQ-9 consists of nine items, scored on a scale from 0 to 3, with a total score ranging from 0 to 27 points, where a higher score indicates a higher level of depressive symptoms. The PHQ-9 has shown strong psychometric properties in a variety of settings [49, 50]. The PHQ-9 score was included as a covariate for the regression analyses, as severe depression may interfere with treatment motivation and adherence [51].

The Patient Exposure and Response Prevention (EX/RP) Adherence Scale (PEAS; [52]) is a self-report questionnaire consisting of three items. The PEAS assesses patient adherence between sessions. The PEAS was completed by patients in the evening of the second and third days. The first item measures the amount of assigned exposures that were attempted, the second item measures the quality of attempted exposures, and the third item measures the degree of success with response prevention. The items are rated on a scale ranging from 1 (none, 0%) to 7 (most, > 90%). The scale has demonstrated good reliability and validity [52]. In this study, we used the mean (across both days) PEAS score as the measure of patient adherence.

### Statistical analyses

There were no differences among the three conditions in the Y-BOCS total score at pretreatment (*p* = .59), posttreatment (*p* = .21) or the 3-month follow-up (*p* = .15). Therefore, we used the total sample for our analyses, testing the associations between treatment motivation and outcomes irrespective of the treatment condition.

As the psychometric properties of the NML2 are questionable, three approaches were taken to secure measures with internal consistency. The first approach involved determining Cronbach’s alphas for the original subscales, and factors with acceptable internal consistency were retained in further analyses. The second approach attempted to represent the NML2 total score by removing items with an item–total correlation below.10. The third approach involved using a MISC scoring procedure to code the 25 NML2 statements according to change talk categories. The items were coded as follows: ability, commitment, desire, need, reason, taking steps, other, or neutral. The Cronbach’s alpha was then used to test for internal consistency in the categories coded. A threshold of Cronbach’s alpha values ≥ .70 was used to define acceptable internal consistency.

Associations between study variables were explored via Pearson correlations. Three hierarchical regression analyses were run to test the association between treatment motivation and treatment outcomes. The analyses controlled for demographic variables in Step 1 and pretreatment severity in Step 2. Motivation was entered in the third step, and adherence was entered in the fourth step. Missing data were not imputed, resulting in a sample size of 148 participants for the regression analyses. Durbin–Watson values (1.7–1.8) indicated no problems with autocorrelation, and the variance inflation factor suggested no problems with collinearity (all values were below 1.19).

Finally, a repeated-measures ANOVA was used to compare changes in the Y-BOCS total score (from pretreatment to posttreatment and the 3-month follow-up) for patients scoring above or below the mean in terms of treatment motivation and adherence. Where the sphericity assumption was violated, corrections were applied via the Greenhouse–Geisser method.

## Results

### Preliminary analyses: assessment of motivation

The three original subscales of the NML-2 had Cronbach’s alphas of.80 (Preparedness subscale, 10 items),.62 (Distress subscale, 5 items), and.67 (Doubt subscale, 5 items). The Preparedness subscale was therefore retained for further analyses, whereas the Distress and Doubt subscales were not.

For the total NML2 score, four items (8, 15, 19, and 23) were removed because of low item‒total correlations. This left 21 items with a Cronbach’s alpha of.78, which were retained for further analyses.

With the MISC approach to the NML2, 13 items with a Cronbach’s alpha of.79 were coded as “Commitment” (see Supplemental Table S1). The NML2 Commitment factor was retained for further analyses. The Commitment scale resembled the Preparedness subscale, but two items from the Preparedness subscale were not included, whereas five were added. Coding using the MISC framework also revealed six items coded as “Reasons”. These items were matched with the Distress items with the addition of one item and had an alpha value of.59. The “Reasons” factor was therefore not included in further analyses. The remaining items were coded as “Need”, “Other”, or “Neutral”, and there were too few items to form a factor.

Therefore, three motivation measures with acceptable internal consistency remained: the NML2 Preparedness subscale score, the NML2 revised total score, and the NML2 Commitment scale score. NML2 Preparedness score, NML2 total score, and NML2 Commitment score were strongly correlated (*r* = .76–.91) reflecting substantial overlap among the three motivation measures employed. There was no difference in the three motivation scores for participants categorized as nonresponders or relapsed patients (*p* = .31,.48, and.54).

### Associations among treatment motivation, treatment adherence, and treatment outcomes

Table 2 summarizes the correlations among the study variables. NML2 total and NML2 subscales scores were not associated with Y-BOCS total score at pre-treatment. The NML2 Preparedness subscale score and the revised total score were not associated with treatment outcomes. The NML2 Commitment score showed a weak significant association with treatment outcomes (*r* = −.19 and −.20), whereas treatment adherence showed a more robust correlation with treatment outcomes (*r* = −.39 and −.42).

**Table 2:** Descriptions and correlations among study variables

	*M* (*SD*)	2	3	4	5	6	7	8	9	10	11
1. Y-BOCS (pretreatment)	27.0 (3.9)	.15	.26**	.08	-.02	.22**	.06	.09	.09	.00	.02
2. Y-BOCS (posttreatment)	12.4 (5.9)	-	.64**	.02	.02	.15	.33**	-.08	-.11	-.20*	-.42**
3. Y-BOCS (3-month follow-up)	13.9 (7.2)		-	.05	.05	.18*	.31**	-.07	-.06	-.19*	-.39**
4. Age	34.6 (10.9)			-	.18	.07	.00	.21**	.09	.17*	-.14
5. Male sex	28.2%				-	-.01	.05	-.04	-.04	-.02	-.19*
6. PHQ-9 (pretreatment)	11.9 (5.9)					-	.08	.10	.15	-.06	.04
7. Y-BOCS (posttreatment; from prior treatment)	14.3 (6.0)						-	-.02	-.05	-.11	-.24**
8. NML2 – Preparedness	45.8 (3.6)							-	.76**	.78**	.17*
9. NML2—Total revised	88.0 (7.9)								-	.91**	.21**
10. NML2 – Commitment	55.3 (5.8)									-	.19*
11. Patient adherence	6.0 (0.7)										-

** < .01; * < .05

*Y-BOCS* Yale–Brown Obsessive Compulsive Scale, *PHQ-9* Patient Health Questionnaire-9, *NML2* Nijmegen Motivation List 2

Adherence showed significant but weak positive associations with the three measures of motivation (*r* = .17–.21). Compared with males, females showed greater treatment adherence. Higher posttreatment Y-BOCS total scores from the prior ERP treatment were associated with lower treatment adherence (*r* = −.24). and worse OCD-treatment outcomes (*r* = .31 to.33). Older age was weakly associated with stronger motivation for two of the three motivation measures (preparedness *r* = .21, commitment *r* = .17).

Three hierarchical regression analyses were conducted to examine the unique contribution of each motivation measure to treatment outcomes, after controlling for demographic variables and pretreatment symptom severity. In all three models, demographic variables (age and gender) were entered in Step 1, and pretreatment Y-BOCS scores in Step 2. In Step 3, one of the three motivation variables was entered: 1) the NML2 Preparedness subscale score, 2) the revised NML2 total score, or 3) the NML2 Commitment score. Treatment adherence was entered in Step 4.

In the first regression analysis, the association between the NML2 Preparedness subscale score and treatment outcomes was tested. In the Step 1, the demographic variables contributed nothing to the explained variance. In Step 2, pretreatment severity explained 12% of the variance and contributed significantly to the explained variance. In Step 3, treatment motivation was not significant, whereas in Step 4, treatment adherence was significant, and contributed to 12% of the variance. In the final step of the equation, treatment adherence and the Y-BOCS total score after the prior ERP treatment were the significant independent variables.

In the second regression analysis, the associations between the revised NML2 total score and treatment outcomes were tested. The first two steps were identical to those of the first regression analysis. In the third step, motivation was not significant, whereas in the fourth step, adherence was significant. In the final step, the results were identical to those of the first regression analysis.

In the third and final regression analysis, the NML2 Commitment score was evaluated. The Commitment score was significant in Step 3, accounting for 3% of the explained variance. However, in the final step, while controlling for all other variables, only treatment adherence and the Y-BOCS total posttreatment score from the the prior ERP treatment were significant. A summary of the regression analyses is provided in Table 3.

**Table 3:** Variables associated with the posttreatment Y-BOCS total score (including three different measures of motivation)

Steps	NML2 Preparedness		NML2 Total		NML2 Commitment	
	*F* cha	*R*^2^ cha	*F* cha	*R*^2^ cha	*F* cha	*R*^2^ cha
Step 1, Demographics	0.2	.00	0.2	.00	0.2	.00
Step 2, Severity	6.4***	.12	6.4***	.12	6.4***	.12
Step 3, Motivation	1.3	.01	2.7	.02	4.4*	.03
Step 4, Adherence	21.5***	.12	20.4***	.11	19.9***	.11

Final step	Adj. *R*^2^ = .21		Adj. *R*^2^ = .21		Adj. *R*^2^ = .22	
	*β*	*t*	*β*	*t*	*β*	*t*
Male sex	-.04	−0.55	-.04	−0.56	-.04	−0.57
Age	-.03	−0.36	-.03	−0.33	-.01	−0.14
Y-BOCS after the prior tx	.22	2.87**	.22	2.87**	.21	2.82**
Y-BOCS (pretreatment)	.10	1.36	.11	1.41	.11	1.41
PHQ-9 (pretreatment)	.14	1.90	.15	1.96	.13	1.78
Motivation	-.02	−0.29	-.06	−0.72	-.10	−1.32
Adherence	-.37	−4.64***	-.36	−4.52***	-.35	−4.46***

Nonimputed data (*N* = 148)

*Y-BOCS* Yale-Brown Obsessive Compulsive Scale, *PHQ-9* Patient Health Questionnaire-9, *NML2* Nijmegen Motivation List 2, *tx* treatment

*** < .001 ** < .01 * < .05

**Fig. 1:**
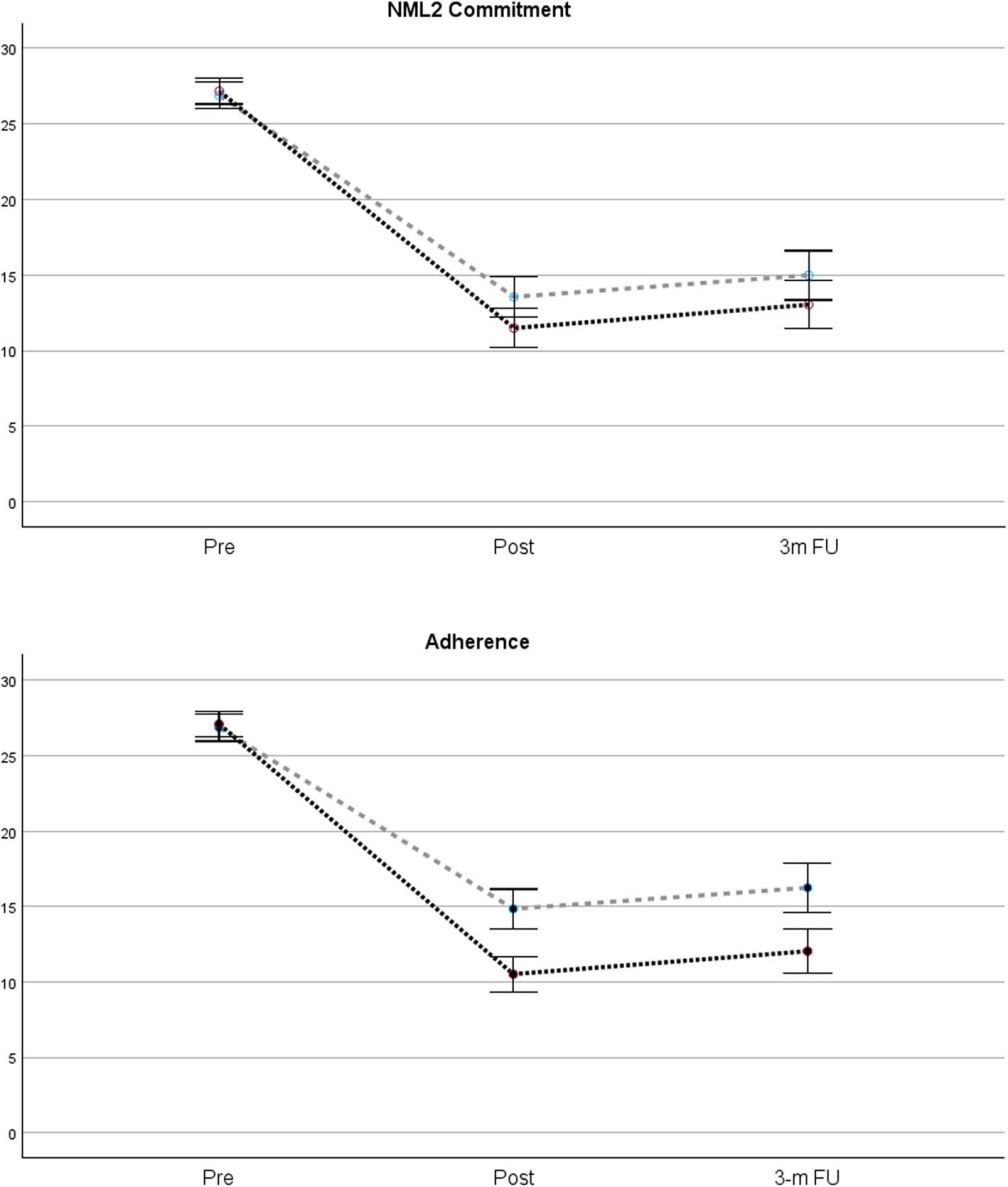
Changes in Y-BOCS total scores for patients with scores above and below the mean adherence and commitment scores Note. The darker lines represent groups with scores above the mean

A visual summary of the associations among commitment, adherence and changes in Y-BOCS total scores is provided in Fig. 1. Both the NML2 Commitment score (*p* = .043) and treatment adherence (*p* < .001) showed a significant interaction with improvement, as patients with scores above the mean for each variable showed greater improvements than those with scores below the mean.

## Discussion

The main objective of this study was to examine the predictive role of treatment motivation in relation to treatment outcomes for patients with treatment-resistant OCD treated with B4DT. There were no clear associations among the NML2 total score, the NML2 Preparedness subscale score, and the treatment outcome. However, the NML2 Commitment score (deducted using the MISC framework) showed some predictive value but not when adherence was controlled for. The lack of a clear predictive validity of self-reported pretreatment motivation is in line with other studies of CBT for OCD [16] and with studies including participants with other anxiety disorders [17, 18].

Consistent with systematic reviews of predictor studies [4–6], our study confirmed that precise predictions based on pretreatment factors are difficult when patients with OCD are treated with CBT. However, this could be good news for patients, as it indicates that most patients have similar potential for recovery.

It is likely that most patients will report high motivation before the start of treatment, as observed in this study, reflected by the mean NML2-item score of 4.2 on a scale ranging from 1–5. This corresponds with the fact that the patients sought help, received a diagnosis, underwent severity assessment, had their substantial distress confirmed, set aside time to attend the 4-day treatment, and received a logical treatment plan for evidence-based treatment. The high motivation could also be related to the RCT research design with patients accepting randomization, medication, and concentrated ERP. However, when treatment starts, patients must face their fears which can challenge their initial motivation. The therapist may address motivational issues during exposure tasks, but not during homework assignments. How a patient experiences these challenges and how the therapist addresses potential setbacks could affect patient motivation throughout therapy. Using motivational interviewing has been suggested as a way of enhancing compliance [53], and motivational interventions provided prior to the start of treatment could enhance treatment outcomes [54, 55]. SSRI treatment could also be a good option for patients who are not motivated to receive ERP treatment.

The observed in-session resistance may be a better predictor of treatment outcomes than pretreatment self-reported motivation [56]. Resistance during sessions typically reflects momentary behaviors such as avoidance, hesitation, or disengagement, which can disrupt therapeutic progress. Persistent resistance may therefore represent a breakdown in adherence within the therapeutic context and may ultimately undermine the efficacy of exposure-based interventions [57]. Motivation likely fluctuates throughout therapy; however, Ponzini et al. [24] reported that there were also trait-like effects of motivation, which could explain the slight predictive effect of pretreatment motivation. It is likely that the predictive value of treatment motivation/adherence increases if it is measured during rather than before treatment [26–28]. Monitoring and intervening in fluctuating motivation may therefore play a role in supporting the therapeutic progress. Treatment responders are expected to show an increasing level of active participation as treatment progresses [28]. However, lower levels of commitment after the first session could be an indicator of lower adherence and poor treatment outcomes [26] but is likely one of many interacting factors.

The fact that the Commitment factor of the NML2 showed the most predictive validity of the NML2 factors could support the statement by Drieschner and colleagues [7] concerning the conceptual ambiguity of motivational measures. Their model proposes that motivation to engage in treatment (MET) predicts actual treatment engagement (TE), defined as the patient’s behavioral participation in therapeutic tasks. However, the association between MET and TE is expected to be modest, as engagement may be influenced by other factors such as cognitive capacity, psychological barriers, or contextual constraints [7]. Our findings are consistent with this distinction. While the NML2 Commitment subscale was more predictive than other motivational indicators, the overall correlation between motivation and adherence during treatment was weak (*r* = .17–.21). This underscores the need to account for additional determinants of adherence beyond initial motivational readiness, and highlights the importance of assessing motivation as a dynamic, process-oriented construct during therapy rather than solely at baseline.

Preliminary findings suggest that a working alliance (a strong therapeutic bond and agreement on tasks and goals) is associated with better treatment adherence [58]. Motivation was also significantly associated with adherence in a previous study but to a lesser extent than working alliances. These findings suggest that there is likely a complex interplay of patient and therapist variables influencing adherence. Future studies using video analyses of treatment should explore how these factors interact and affect adherence in detail, as therapists probably play a pivotal role in maintaining patients’ treatment motivation. Additionally, using more treatment-specific motivational measures could yield different findings from more generic psychotherapy measures of motivation. Using OCD- and CBT-specific measures of treatment readiness (targeting readiness to stop rituals/compulsions, not avoiding triggering situations, and attitudes toward exposure and response prevention) could provide more precise measures and have stronger associations with adherence [59]. Specific factors may be more important than common factors in OCD treatment [60], and the most important factors are exposure and response prevention [61]. However, common factors are likely an important part of how patients and therapists navigate in dealing with the exposure exercises.

If a therapist reacts in a nonflexible manner while treating patients with difficult-to-treat conditions who struggle with exposures, it could make matters worse. A study showed that therapist (not patient) adherence and patient motivation could interact, as greater therapist protocol adherence was associated with poorer outcomes for less motivated patients [62]. The authors discussed this finding as illustrating the importance of therapists identifying potential difficulties early and addressing them flexibly within the boundaries of the treatment plan. Importantly, however, their assessment of motivation only consisted of one item rated by therapists.

This study has several limitations that must be considered when interpreting the results. The NML2 has previously been found to predict dropout. However, attrition analyses could not be conducted in this study, as only one patient dropped out. This suggests that the intensive treatment format is associated with low attrition rates. As dropout was minimal, it would have been interesting to compare treatment motivation among potential participants before seeking treatment. Treatment motivation could differ between treatment seekers and nonseekers (or refusers). However, this study lacked data on nonparticipants. The study used the NML2, which has psychometric and conceptual issues, and different versions have been used in previous studies. Therefore, this study tested different versions of the NML2 to obtain factors with acceptable internal consistency. Further psychometric and conceptual investigations of the NML2 would be beneficial. Repeated measures of treatment motivation and different approaches to assessing motivations would also be beneficial. The NML2 could also have ceiling effects, as the patients had a mean item score of 4.2 points on a scale ranging from 1–5. Given the psychometric quality of the NML2, the results regarding motivation may not be reliable.

Taken together, the findings suggest that treatment adherence was more strongly associated with treatment outcomes than pretreatment self-reported motivation. Treatment motivation and working alliance are likely associated with treatment adherence and may therefore be important targets for future research. Future research should investigate the effects of therapist behaviors on patients’ treatment motivation and adherence.

## Supplementary Information

Supplementary Material 1.

## Data Availability

The data that support the findings of this study are not openly available due to reasons of sensitivity and are available from the corresponding author upon reasonable request. The data are stored in a controlled access database at Haukeland University Hospital.
