# Supplementary material for: Motivation in the Bergen 4-day treatment for obsessive–compulsive disorder
Source: BMC Psychiatry. 2025 Oct 9;25:958. doi: 10.1186/s12888-025-07218-z (PMC12512304; doi:10.1186/s12888-025-07218-z)
Supplement: Supplementary file 1 — Supplementary Material 1. [file 12888_2025_7218_MOESM1_ESM.docx]

Supplemental Table

**Table S1**

*Misc scoring of NML2*

| NML2 items | Misc code |
| --- | --- |
| 1. My problems make me profoundly unhappy. | Reason |
| 2. I will do anything to get rid of my problems. | Commitment |
| 3. I do not believe that this is the right treatment for me*. | Commitment |
| 4. I urgently need help in solving my problems. | Need |
| 5. I’m certain that I shall also practice at home the things I learn in treatment | Commitment |
| 6. I expect to benefit more from therapy if I actively participate in it. | Commitment |
| 7. I’m willing to put work or other activities aside in order to attend treatment sessions. | Commitment |
| 8. My problems make me feel ashamed. | Reason |
| 9. I keep my appointments, no matter what. | Commitment |
| 10. I’m not very optimistic about the outcome of the treatment I’m about to begin*. | Commitment |
| 11. I’m prepared to work on myself for a while. | Commitment |
| 12. I think I’m difficult to treat*. | Commitment |
| 13. I’m willing to postpone other appointments to attend treatment. | Commitment |
| 14. I made the right decision in attending therapy. | Other |
| 15. My problems make me a nuisance to others. | Reason |
| 16. I do not know whether I’ll find sufficient time to carry out homework assignments well*. | Commitment |
| 17. My problems will disappear of their own accord*. | Other |
| 18. My problems do not bother me*. | Reason |
| 19. I can’t help having problems*. | Follow/Neutral |
| 20. I think it’s a nuisance having to carry out homework assignments as well*. | Commitment |
| 21. I believe that this treatment will help me get rid of my problems. | Commitment |
| 22. Other people notice that I’m functioning less well. | Reason |
| 23. I’m known as someone who perseveres. | Follow/Neutral |
| 24. I don’t get much support from those around me*. | Follow/Neutral |
| 25. Despite my problems, I can function well in daily life*. | Reason |

*Note*. * = reversed items. Items 8, 15, 19, and 23 were not included in the revised total score. Preparedness items = Items 2, 4, 5, 6, 7, 9, 11, 13, 14, and 21.
